# Paraneoplastic Recurrent Hypoglycaemic Seizures: An Initial Presentation of Hepatoblastoma in an Adolescent Male—A Rare Entity

**DOI:** 10.1155/2014/104543

**Published:** 2014-11-30

**Authors:** Irappa Madabhavi, Apurva Patel, Mukesh Choudhary, Suhas Aagre, Swaroop Revannasiddaiah, Gaurang Modi, Asha Anand, Harsha Panchal, Sonia Parikh, Shreeniwas Raut

**Affiliations:** ^1^Department of Medical and Paediatric Oncology, GCRI, Ahmedabad, Gujarat 380016, India; ^2^Department of Radiotherapy, Government Medical College, Haldwani, India

## Abstract

Hepatoblastoma (HB) is a rare malignant tumour of the liver and usually occurs in the first three years of life. Hepatoblastoma in adolescents and young adults is extremely rare; nevertheless the prognosis is much worse than in childhood, because these kinds of tumours are usually diagnosed late. Characteristic imaging and histopathological and AFP levels help in the diagnosis of hepatoblastoma. Paraneoplastic features of hepatoblastoma are not uncommon at presentation and include erythrocytosis, thrombocytosis, hypocalcaemia, isosexual precocious puberty, and rarely hypoglycaemia. Even though hypoglycaemia is commonly seen in hepatocellular carcinoma, its association with hepatoblastoma is very rare. We present a case of 15-year-old male patient presenting with complaints of recurrent hypoglycaemic seizures ultimately leading to diagnosis of hepatoblastoma. Managed successfully with neoadjuvant chemotherapy, surgery and adjuvant chemotherapy with adriamycin and cisplatin based regimens. An extensive review of literature in the PubMed and MEDLINE did not reveal much data on paraneoplastic recurrent hypoglycaemic seizures as an initial presentation of hepatoblastomas in adolescents and young adults.

## 1. Introduction

Hepatic tumours represent approximately 0.5–2% of all the tumours in children and are responsible for 1–4% of all the solid tumours. Approximately 90% of the cases occur in patients under 5 years of age and two- thirds of the cases occur in the first 2 years of life. In adolescents and adults, hepatoblastomas are extremely rare, and the initial symptoms are nonspecific and the usual presentation is failure to thrive, loss of weight, and a rapidly enlarging abdominal mass.

Paraneoplastic features of hepatoblastoma are not uncommon at presentation and include erythrocytosis, thrombocytosis, hypocalcaemia, isosexual precocious puberty, and rarely hypoglycaemia. About 10–30% of hepatic cancer may lead to hypoglycaemia. Plasma insulin, C-peptide, and proinsulin levels are low and free IGF-II levels will be elevated during hypoglycaemia.

The initial diagnosis of HB is mainly based on imaging, histopathology, and AFP levels. The complete surgical resection is the cornerstone of treatment; however, the tumour is often unresectable at the time of diagnosis. Chemotherapy has been proven effective in both an adjuvant and neoadjuvant treatment and can shrink tumours. AFP level is a valuable tumour marker to see response of presurgical chemotherapy, in the evaluation of the excision result and for the precocious diagnosis of the hepatoblastoma relapse.

## 2. Case Report

Our patient was an otherwise healthy 15-year-old adolescent male who was referred to us from a local physician. The patient initially presented with giddiness and syncope for three weeks followed by recurrent multiple episodes of abnormal body movements, associated with frothing from the mouth, and postictal confusion. These symptoms used to subside with glucose tablets and local made glucose solutions. In view of the classical history of seizures and drastic improvement in the symptoms with glucose solutions, patients serum was sent for glucose level estimation and it was found to be 17 mg/dL. Patient was investigated for severe hypoglycaemia with serum insulin levels, C-peptide levels, fasting blood sugar, and postprandial blood sugar levels which were within the normal range. Ultrasonography of the abdomen was showing hepatomegaly for which he has been referred to us for further management. There was no history suggestive of diabetes or oral hypoglycaemic drug intake.

On clinical examination his height, weight, body mass index, blood pressure, pulse rate, and respiratory rate were within the normal range for his age and sex. There was no icterus, pallor, lymphadenopathy, or any other signs of liver disease. On per abdominal examination he was found to have moderate hepatomegaly, not associated with any bruit or venous hum.

His blood investigations revealed normal complete blood counts, renal function tests, and liver function tests. His serum sample was negative for hepatitis B surface antigen, antihepatitis B antibody, and antihepatitis C antibody. Alpha-fetoprotein (AFP) level was 155920 ng/Ml. In view of symptomatic hypoglycaemic episodes with normal insulin, C-peptide levels, patient's serum was sent for free insulin-like growth factor-II (IGF-II) levels which showed very high levels.

**Figure 1:**
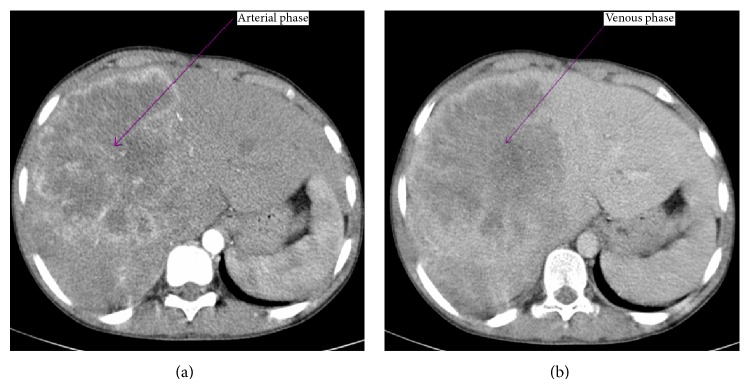
Ill-defined soft tissue density lesion of size 12 × 10 cm in right lobe of liver which shows marked heterogeneous enhancement on arterial phase (a) and shows washout of the contrast in the venous phase (b).

**Figure 2:**
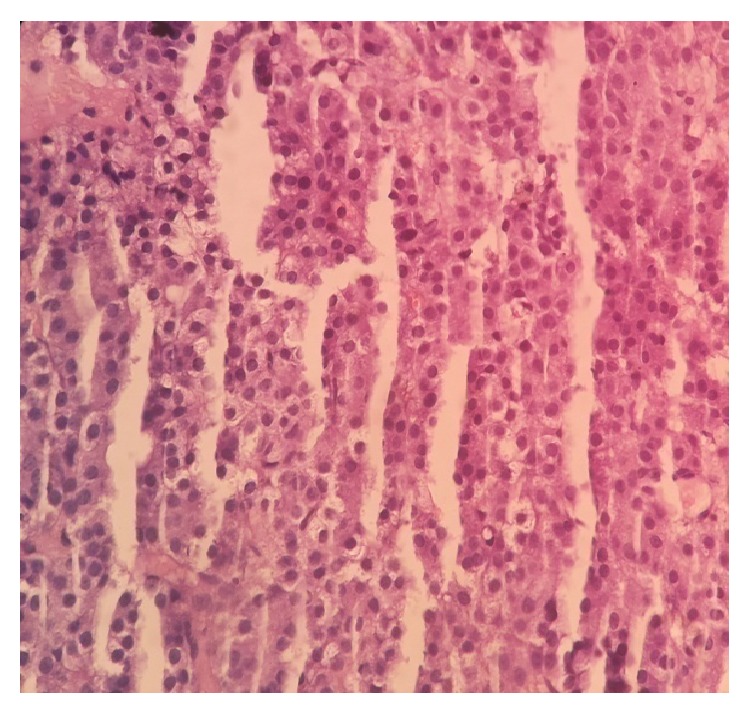
Atypical cells arranged in 1-2 layers of trabeculae separated by sinusoids. Individual cells are having round nucleoli with moderate granular cytoplasm.

Computed tomography (CT) of abdomen shows an ill-defined soft tissue density lesion of size 12 × 10 cm in right lobe of liver which shows marked heterogeneous enhancement on arterial phase (Figure 1(a)) and washout of the contrast in the venous phase (Figure 1(b)). USG-guided biopsy sample was obtained with an 18 G tru-cut needle. Histopathology examination of the biopsy specimen shows atypical cells arranged in 1-2 layers of trabeculae separated by sinusoids. Individual cells are having round nucleoli with moderate granular cytoplasm and some cells showing clear cytoplasm. Overall findings were suggestive of foetal type of hepatoblastoma (Figure 2).

**Figure 3:**
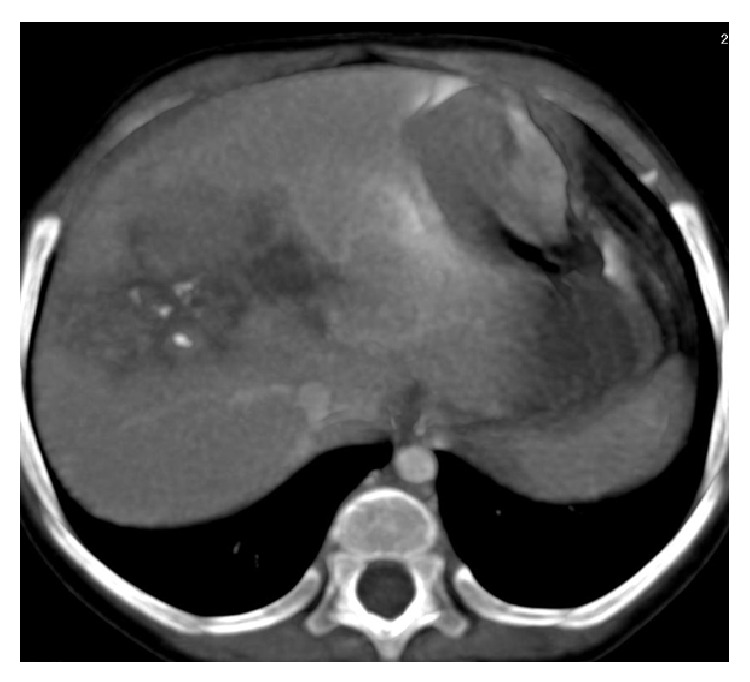
Postchemotherapy CT image shows a heterogeneously enhancing soft tissue density lesion of size 45 × 65 mm, with internal foci of calcification involving right lobe of liver.

After the results of biopsy, four cycles of neoadjuvant chemotherapy with adriamycin 25 mg/m^2^/day for 3 days and cisplatin 20 mg/m^2^/day for 5 days were given. After two cycles of chemotherapy there was significant improvement in hypoglycaemic episodes. Neoadjuvant postchemotherapy abdominal CT image shows a heterogeneously enhancing soft tissue density lesion of size 45 × 65 mm, with internal foci of calcification involving right lobe of liver, suggesting a partial response as compared to prechemotherapy image according to RECIST criteria (Figure 3). There was a 2-log reduction in AFP levels. A right partial hepatectomy was performed without any postprocedural complications. Postoperative adjuvant systemic chemotherapy with adriamycin and cisplatin was continued for another 4 cycles. Posttreatment imaging study did not show any residual lesion and AFP levels were within normal limits. Patient is under regular followup with 3 monthly ultrasound abdomen and AFP levels in our centre since one year without any abnormal findings.

## 3. Discussion

Hepatic tumours represent approximate 0.5–2% of all the tumours in child and are responsible for 1–4% of all the solid tumours. Hepatoblastoma represents the most common malignant hepatic tumour in childhood. Most cases of hepatoblastoma are sporadic; however, it might be associated with Beckwith-Weidman syndrome, familial adenomatous polyposis (FAP) coli, low birth weight, and genetic syndromes.

Hepatoblastoma (HB) is a rare malignant tumour of the liver and usually occurs in the first three years of life [1]. Approximately 90% of the cases occur in patients under 5 years of age and two-thirds of the cases occur in the first 2 years of life [2]. Most of these tumours arise in the embryo; hence it seems to be unusual that hepatoblastoma occurs in adolescents. HB in adolescents and young adults is extremely rare; nevertheless the prognosis is much worse than in childhood, because these kinds of tumours are usually diagnosed late. Since Bartok in 1958 described the first hepatoblastoma case in an adult patient, about 45 cases have been reported in the literature [3, 4].

The aetiology of HB has been elusive. Present investigations of the cytogenetic and molecular genetic abnormalities in HB revealed involvement of chromosomal loci on 1q, 2 (or 2q), 4q, 8 (or 8q), and 20. Loss of heterozygosis imprinting at locus 11p 15.5 also suggests a common genetic basis for HB [5]. The detection of nuclear *β*-catenin accumulation implies an oncogene alteration of the wnt/*β*-catenin pathway. Furthermore, nuclear p53 accumulation indicates that p53 mutation is also involved in the molecular pathogenesis of the malignancy [6].

Histologically, hepatoblastomas may present in two variants: (a) the epithelial type, which consists of foetal and embryonic cells presenting alone or in combination; (b) the epitheliomesenchymal mixed type, in which mesenchymal elements are present along with the epithelial component. It has been thought that hepatoblastoma develops during intrauterine life, but the same histological pattern has been seen in hepatic tumours in adults.

Hepatoblastoma usually develops in the right hepatic lobe. The left hepatic lobe receives oxygenated blood from the umbilical vein, while the right lobe receives oxygenated blood from the portal vein, with lower oxygen saturation. The lower oxygen saturation could favour the embryonic differentiation of the hepatoblastoma in certain conditions, this explaining the more frequent localization in the right hepatic lobe.

In adolescents and adults, the morbidity of HB is extremely rare, and the initial symptoms are nonspecific and the usual presentation is failure to thrive, loss of weight, and a rapidly enlarging abdominal mass. Systemic symptoms are rare and very rarely symptoms of hypoglycaemia may be present, such as in our case. The serum AFP level is almost invariably high [7].

Paraneoplastic features of hepatoblastoma are not uncommon at presentation and include erythrocytosis, thrombocytosis, hypocalcaemia, isosexual precocious puberty, and rarely hypoglycaemia [8]. Our patient had history of recurrent hypoglycaemic seizures as the only sole manifestation of the symptom complex leading to diagnosis of hepatoblastoma in an adolescent male.

Nonislet cell tumour induced hypoglycaemia has been reported with tumours of mesenchymal, epithelial, or haemopoietic origin. Hepatic tumour and gastric and lung cancer are common epithelial cancers that can cause hypoglycaemia. Hypoglycaemia disappears with definitive treatment of nonislet cell neoplasm.

The definition of the hypoglycaemia is defined as blood glucose levels less than 54 mg/dL. The metabolism in brain almost completely depends upon oxygen and glucose; when brain is deprived of the glucose, patients would show a series of neurological and psychological disorders. The mild symptoms are lack of concentration, behaviour changes, dizziness, vertigo, blurred vision, hallucination, and tics. When these symptoms get worse, patients develop convulsion and may fall into a coma or even die.

About 10–30% of hepatic cancer may lead to hypoglycaemia [9, 10]. Two types of hypoglycaemia (Types A and B) have been described in hepatic tumour. Type A occurs in terminal stages, especially in large tumours, when the energetic metabolism of the cancer cells would consume large amount of glucose and the glycogen storage would be seriously deficient, so it is hard to maintain glucose stability. And abnormalities in liver function cause the metabolism of insulin in liver to slow down and the function time of insulin prolongation, which results in the fact that liver could not convert nonglucose substances into glycogen and the ability of glycogenesis is reduced.

Type B occurs in 5% of the cases and is early in the course of the disease due to increased production of IGF-II by the tumour suggesting a paraneoplastic manifestation. Overproduction of insulin-like growth factor II (IGF-II) specifically as incompletely processed does not complex normally with circulating binding proteins and thus more readily bind to target tissue, is the cause of hypoglycaemia in most patients. Plasma insulin, C-peptide, and proinsulin levels are low and free IGF-II levels will be elevated during hypoglycaemia. Growth hormone and IGF-I concentration will be reduced due to negative feedback mechanism mediated by IGF-II [11].

Other reports found that in hepatic tumour patient with paraneoplastic syndromes, both the AFP and tumour size are higher than those without paraneoplastic syndromes. The possible hypothesis to explain it is that hepatic tumour patients with paraneoplastic syndromes have higher level of serum AFP and this is mainly because of the overexpression of AFP gene in hepatic cells which leads to the increase of serum AFP value. Meanwhile, tumour cells stimulate the biosynthesis of hormone like substances, such as IGF-II, parathyroid hormone-related protein (PTHrP), and erythropoietin, which leads to the expression of paraneoplastic syndromes such as hypoglycaemia, hypocalcaemia, and erythrocytosis [12].

The initial diagnosis of HB is mainly based on imaging. Proper diagnosis, staging, and treatment of HB require accurate imaging studies like ultrasound (US), computed tomography (CT), and magnetic resonance imaging (MRI). Other standard investigation includes serum AFP. However, the final diagnosis relies on tumour biopsy.

The complete surgical resection is the cornerstone of treatment for patients with HB and is the only chance of an optimal clinical result; however, the tumour is often unresectable at the time of diagnosis. Chemotherapy has been proven effective in both an adjuvant and neoadjuvant treatment and can shrink tumours. It makes them less prone to bleed and delineates the tumour from the surrounding normal parenchyma and vascular structures so as to facilitate the resections. HB is sensitive to such chemotherapy drugs as doxorubicin, cisplatin, vincristine, 5-FU, and cyclophosphamide [13].

There are two different strategies regarding the treatment of HB. The SIOPEL Group recommends the preoperative chemotherapy followed by the tumoral excision and then postoperative chemotherapy [14]. The American study group consider the surgical intervention when diagnosed (applicable to 50% of the patients) followed by postsurgical chemotherapy [15].

The SIOPEL established the PRETEXT (pretreatment tumour extension) staging system, reflecting the number of liver sections with or without tumour, that is,

 PRETEXT I: three adjoining liver sections free, one section involved;
 PRETEXT II: two adjoining sections free, two sections involved;
 PRETEXT III: two nonadjoining sections free or just one section free, in the latter case three sections involved;
 PRETEXT IV: no free section, all four sections involved.

The aim of the PRETEXT classification is to assess the operability prior to any treatment. This approach has shown reliable interobserver reproducibility and an excellent prognostic value. Its main limitation is the difficulty in accessing between actual invasion of a liver segment and displacement of an anatomical border [16].

PRETEXT I–III tumours are treated with partial hepatectomy and PRETEXT IV or unifocal, centrally located tumours with total hepatectomy, that is, liver transplantation [17]. Hepatoblastoma is considered to be unresectable, when the tumour is extremely large, involving the risk of severe haemorrhage; when both hepatic lobes are altered; when the hepatic vein or the inferior vena cava is affected. Gold standard chemotherapy—popularized by SIOPEL with acronym PLADO (cisplatin and doxorubicin).

Lung metastases are not an absolute contraindication to liver resection or even liver transplantation. Pulmonary metastases should be removed first either by surgery or by chemotherapy and then subsequently primarily tumour-resected—either by partial hepatectomy or by liver transplantation. The hepatic transplant is indicated either initially for the unresectable hepatoblastoma or after the relapse.

AFP level is a valuable tumour marker to see response of presurgical chemotherapy, in the evaluation of the excision result and for the precocious diagnosis of the hepatoblastoma relapse [18]. The complete excision of the hepatoblastoma determines the decrease of the AFP serum level, which will be normalized after 4–6 weeks. The persistency or secondary increase of the AFP values represents a residual tumour, metastases, or a relapse. The prognosis of patients with HB varies with the histology and stage. The favourable outcome associated with pure foetal histology and the poor prognosis of anaplastic (small-cell undifferentiated) hepatoblastomas.

Because of the lack of experience of hepatoblastomas in adolescent and adult patients, tumours can be treated with surgical resection (if it is possible) and chemotherapy as in children. However, mean survival time in adult patients is 3.5 months. Liver transplantation has recently been associated with significant success in the treatment of children with unresectable hepatic tumors.

## 4. Conclusion

Hepatoblastoma in adolescent and adult patients has an aggressive presentation and a poor prognosis compared to childhood patients. The symptoms of hepatic tumours patients with complicated hypoglycaemia tend to be erroneously diagnosed as central nervous system diseases, intracranial metastases of tumours, or hepatic encephalopathy. So when giving clinical treatments to hepatic cancer patients, it is necessary to pay attention to the change of their blood glucose levels, especially for those patients who have fallen into a coma.

## 5. Learning Points

1. Hepatoblastoma (HB) is a rare malignant tumour of the liver and usually occurs in the first three years of life.
2. Characteristic imaging and histopathological and AFP levels help in the diagnosis of hepatoblastoma.
3. Paraneoplastic features of hepatoblastoma are not uncommon at presentation and include erythrocytosis, thrombocytosis, hypocalcaemia, isosexual precocious puberty, and rarely hypoglycaemia.
4. Chemotherapy has been proven effective in both an adjuvant and neoadjuvant treatment and can shrink tumours.
5. HB is sensitive to such chemotherapy drugs as doxorubicin, cisplatin, vincristine, 5-FU, and cyclophosphamide.
6. The complete surgical resection is the cornerstone of treatment for patients with HB and is the only chance of an optimal clinical result; however, the tumour is often unresectable at the time of diagnosis.
